# Endometrial immune cell profile at the time of frozen embryo transfer as prognostic indicator of live birth

**DOI:** 10.3389/fimmu.2026.1719211

**Published:** 2026-03-06

**Authors:** Suset Rodriguez, Laura Padula, Eva Fisher, Bria Slater, Sujad Younis, Carmel Awadallah, Aryana Mohtasham Gharagozloo, Roberta Carlotta Rubino, Mohammed Ibrahim, Michael Paidas, Natasa Strbo, George Roshdy Attia

**Affiliations:** 1 Division of Reproductive Endocrinology, and Infertility, Department of Obstetrics, Gynecology and Reproductive Sciences, University of Miami Miller School of Medicine, Miami, FL, United States; 2 Department of Microbiology and Immunology, University of Miami Miller School of Medicine, Miami, FL, United States

**Keywords:** embryo transfer, immune cells, implantation, IVF, live birth, neutrophils, γδ T cells

## Abstract

**Introduction:**

Endometrial receptivity is essential for implantation in both natural and ART cycles, yet the cellular and molecular environment of the endometrium during this window remains poorly characterized. While cytokines affecting implantation have been studied, data on immune cell subtypes in the endometrium are limited. The objective of this study was to determine the association between endometrial immune cell profile at the time of transfer and live birth in patients undergoing frozen embryo transfer (FET) using the index cycle.

**Methods:**

This exploratory prospective observational cohort study (IRB#20190139) included 48 patients undergoing a hormone replacement FET cycle between May 2022 and May 2024. After ultrasound-guided FET, the catheter tip was rinsed in IMDM + 10% FBS, centrifuged, and stained for CD45, CD3, CD19, CD4, CD8, γδ TCR, CD25, CD127, CD66b, CD14, CD16, and CD56. The primary outcome was live birth. Secondary outcomes included miscarriage, biochemical pregnancy, and ectopic pregnancy.

**Results:**

Elective single embryo transfer was performed for all the patients. There were 24 live births (50%), four miscarriages (8.3%), and three biochemical pregnancies (6.3%). There was no significant difference in demographics of patients that had a live birth compared to those who did not achieve implantation. There was an increased percentage of γδ T cells in the group of live birth compared to non-pregnant group (p=0.019). In contrast, an increased percentage of neutrophils (CD66b^+^) was noted in patients that did not achieve implantation (p<0.003). Importantly, we found receiver operating characteristic (ROC) curve area under the curve (AUC) of 0.72 with 95% confidence interval (CI) 0.5504 to 0.8989 for γδ T cells and AUC is 0.75 (95% CI 0.5681 to 0.9319) for CD66b^+^ cells, confirming the overall ability of these two tests to discriminate between patients who will achieve a live birth vs. ones who will have failed implantation.

**Discussion:**

Our findings suggest that the uterine immune environment during FET may be associated with implantation outcomes. Characterization of endometrial immune cell profiles could provide insights into biological factors linked to implantation and live birth, although their clinical utility remains to be determined. To our knowledge, this study is among the first to describe associations between immune cell profiles assessed during the index FET cycle and subsequent IVF outcomes, supporting a potential role for endometrial immune composition in pregnancy success.

## 1 Introduction

A successful early pregnancy arises from the coordinated interplay of maternal, paternal, and embryonic factors, which together shape the immunological and physiological environment required for implantation and placental development (1, 2). These components must therefore be considered in an integrated manner when examining mechanisms of early gestational success or failure. The decidua contains a highly heterogeneous and functionally specialized immune cell compartment, with leukocytes comprising approximately 40–50% of all decidual cells during the first trimester (3, 4). These immune populations participate in a broad array of essential processes, including maternal–fetal tolerance, tissue remodeling, angiogenesis, and regulation of trophoblast invasion (5, 6). Their balanced activity is critical for establishing a receptive implantation site and promoting proper placental morphogenesis.

*In vitro* fertilization (IVF) and embryo transfer (ET) has become the mainstay treatment option for patients with infertility. Over the years, in spite of the advancement in laboratory technology and different strategies to refine stimulation and embryo transfer protocols, cycle outcomes and pregnancy rates showed slight improvement (7). Some of the recent efforts to improve cycle outcomes include the introduction of preimplantation genetic testing for aneuploidy (PGT-A) to select a euploid embryo for transfer, however this procedure carries risks including embryo harm, inaccurate results, loss of viable embryos, added financial/emotional strain, and lack of consistent evidence for improving live birth rates (8). More recently the introduction of the embryoscope was thought to improve IVF success by allowing for uninterrupted monitoring of embryo development however a recent Cochrane review concluded no clear benefit to improve live birth compared to standard incubation methods (9).

Given these limitations in embryo-focused strategies, recent attention has shifted toward understanding the endometrial environment as a critical factor influencing implantation and overall IVF success. While some markers have been considered to identify the optimal endometrial environment for implantation, such as tumor necrosis factor, cell adhesion molecules, and cadherin, there has been limited clinical applicability (5, 10). In attempts to improve pregnancy rates after IVF, the endometrial receptivity analysis (ERA) aims to identify a personalized window of implantation using microarray technology but it’s validity has been questioned in recent studies (11).

In recent years, the role of endometrial immune cells in reproductive success has garnered significant attention in the field of reproductive immunology and assisted reproductive technology to increase cycle success. Endometrial immune cells, particularly decidual natural killer (dNK) cells, T regulatory cells (Tregs), macrophages, and dendritic cells, play key roles in implantation and early pregnancy. Within this immune landscape, natural killer (NK) cells belong to the innate lymphoid cell (ILC) family, specifically the group 1 ILC subset, and play a dominant role during early pregnancy (12–16). Several groups have characterized the unique properties of dNK cells and have highlighted the pivotal role of dNK cells as “master regulators” of pregnancy (17–20). dNK cells are phenotypically and functionally distinct from peripheral NK cells, displaying reduced cytolytic capacity and instead adopting regulatory and trophic functions that support spiral artery remodeling, angiogenesis (17), and controlled trophoblast invasion (21, 22). This shift from cytotoxicity to tissue-supportive activity is a hallmark of the maternal–fetal interface.

Additionally, studies by Kofod et al. and Lédée et al. suggest that the composition of immune cells in the endometrium can predict reproductive success, with specific immune profiles correlating to higher live birth rates following IVF (23, 24). Higher levels of CD57^+^ NK cells and optimal IL-15/Fn-14 ratios were associated with better pregnancy outcomes (23). However, these studies required endometrial biopsy, preventing embryo transfer in the same cycle. Additionally, they faced challenges related to variability between cycles and inconsistencies in inherited endometrial characteristics.

Our observational study addresses the challenges of assessing the optimal timing of embryo transfer due to the disruption caused by traditional endometrial biopsies, which prevent embryo transfer within the same cycle and may not provide relevant information for future cycles. As a noninvasive alternative, we focus on sampling endometrial fluid at the index cycle, which has shown promise in assessing the endometrial environment and predicting implantation potential (17, 18, 23–25). Previous data, including our own, demonstrate that immune cells are present in uterine fluid during frozen embryo transfer (FET), and the composition of these immune cells may influence pregnancy outcomes (26).

This study seeks to examine the relationship between endometrial immune cell profiles and live birth outcomes using uterine fluid samples collected during the index embryo transfer cycle. Building on prior work, we aim to characterize immune features associated with pregnancy outcomes and to generate hypotheses regarding the role of endometrial immune composition in implantation success. While these findings may inform future studies, additional validation will be required to determine whether immune profiling has relevance for clinical stratification or therapeutic intervention in assisted reproductive technology (ART).

In the long term, and acknowledging the exploratory nature of this study, these findings may help inform the development of tools to more efficiently characterize endometrial immune features using uterine fluid samples obtained during the embryo transfer cycle. The development of a point-of-care test (POCT) capable of rapidly characterizing key endometrial immune signatures directly from uterine fluid samples collected during the embryo transfer cycle, would ideally quantify clinically relevant immune parameters—including the relative abundance or activation state of decidual NK cells, macrophage subsets, T cell populations, and inflammatory mediators—using rapid molecular profiling techniques (e.g., microfluidic immunoassays, multiplex cytokine detection, or portable flow-cytometric/ILC-profiling platforms). However, substantial methodological development and prospective validation will be required to determine feasibility, interpretability, and any potential relevance to clinical workflows.

## 2 Materials and methods

### 2.1 Study design and subjects

The University of Miami Institutional Review Board (IRB) approved this study (#20190139). Participants were included only after providing written informed consent. The study was conducted as a prospective observational cohort from May 2022 to May 2024 at a single academic-based fertility center. Resource limitations and reduced personnel availability contributed to extended sample collection periods. Eligible participants were patients aged 18 to 52 years who had undergone an IVF cycle resulting in at least one frozen embryo available for transfer, regardless of the IVF indication. Patients with uterine pathology or those with cancelled cycles due to failure of embryo thaw survival were ineligible to participate.

A total of 48 participants were recruited after providing written informed consent. Endometrial preparation followed the institution’s hormone replacement treatment protocol, starting with oral estradiol on day 3 of the cycle, which was incrementally increased with transdermal estradiol, followed by vaginal estradiol. Intramuscular progesterone in oil was then administered for luteal phase support. Patients were monitored via transvaginal ultrasound (US) throughout the transfer cycle at preference of the provider. The FET was then performed after 6 days of progesterone administration. All embryos transferred were day 5 or 6 blastocysts. A pregnancy test was performed 10–12 days after the FET. Beta hCG monitoring was performed per institution protocol and serial weekly ultrasounds were performed to monitor the pregnancy starting at 6 weeks gestation until 9 weeks gestation.

Implantation was defined by a positive beta hCG serum measurement taken 10–12 days after the FET. The hCG threshold used to classify samples as positive is greater than 2 mIU/mL. The assay detection limit is 0.1 mIU/mL. Live birth was defined as the delivery of one or more live-born infants. Biochemical pregnancy was defined as a pregnancy identified solely through the detection of rising beta hCG on serum test without evidence of a gestational sac on US. Miscarriage was defined as the loss of pregnancy less than 20 weeks gestation. Ectopic pregnancy was defined as the implantation of a gestational sac outside the uterine cavity.

### 2.2 Sample acquisition and processing

The transfer catheter (Rocket Medical EchoCath™ Embryo Transfer Catheters, U.K.) was used to perform the embryo transfer under US-guidance. The embryo was placed in the uterus approximately 1–2 cm from the fundus. Upon removal of the catheter from the uterus and after catheter verification by the embryologist, the tip of the inner catheter was rinsed externally in 15 ml tube with 5 ml of Iscove’s Modified Dulbecco’s Medium (IMDM) with 10% fetal bovine serum (FBS) (reagents from Gibco, Billings, Montana, U.S.). The outer sheath was not used for the purpose of the study. The specimen was then placed on ice and transferred to the laboratory for analysis within 30 minutes.

The 15 ml tube (Tube 1), containing tip of the catheter in 5 ml of IMDM + 10% FBS, was first vortexed for 1 minute (3400 rpm) and then centrifuged for 10 minutes at 1200 rpm (250 g). After centrifugation, the catheter was carefully removed without disturbing the pelleted cells and put in another empty 15 ml tube (Tube 2) and then the catheter was flushed with IMDM to make sure all of the cells are rinsed. Tube 2 was then centrifuged for 10 minutes at 1200 rpm (250 g), and the cell pellets (from Tube 1 and Tube 2) were combined and proceeded to count. We used automated cell counter to get the number of live cells per ml. A refrigerated centrifuge (+ 4^0^ C) was always used to spin the samples.

Cells were first labeled with live/dead detection kit according to manufacturer instructions (Thermo Fisher Scientific, Waltham, MA, USA) and then resuspended in Human BD Fc Block™ Reagent, (BD Bioscience) for 5 min at room temperature prior to staining with a surface-stain cocktail containing the following antibodies purchased from BioLegend^®^ (San Diego, CA, USA): APC/Cy7 anti-human CD45/Clone HI30, Brilliant Violet 605 anti-human CD19/Clone HIB19, APC anti-human CD4/Clone GK1.5, Spark Blue 550 anti-human CD8/Clone 53-6.7, Brilliant Violet 421anti-human γδ TCR/Clone B1, PercP/Cyanine5.5 anti-human CD25/Clone BC96, Brilliant Violet 510 anti-human CD127/Clone A019D5, PE anti-human CD66b/Clone HCD56, PE/Cy7 anti-human CD14/CloneM5E2, Pacific Blue anti-human CD16/Clone 3G8, PE/Dazzle 594 anti-human CD56/Clone HCD56. Alexa Fluor 700 anti-human CD3/Clone SP34–2 was purchased from BD Pharmingen (San Diego, California, US). Antibody dilutions used ranged from 1:100-1:200; (a final amount of 1µg of the fluorescent antibody per million of cells). After 30 min, cells were washed with a flow cytometry staining buffer (FACS buffer), centrifuged for 5 minutes at 2,500 rpm and then fixed with 2% paraformaldehyde.

Data were collected on Spectral analyzer SONY SP6800 instrument (Sony Biotechnologies, Inc, San Jose, CA, USA), acquiring at least 100,000 total events. We utilized Sony software with a spectral unmixing wizard to guide the process of spectral unmixing (this is a mathematical algorithm used in spectral flow cytometry to separate overlapping fluorescent signals from multiple fluorochromes within a single sample). Single-stained controls were prepared to create the spectral library, and gates were set on these single-color reference controls, focusing on the brightest populations to capture the pure spectral signature of each fluorophore. Unmixing matrix was loaded into data analysis software to apply to all experimental samples. We also used negative staining controls as well as fluorescence minus one (FMO).

The analysis was conducted using FlowJo 10 software (Tree Star). Initially, cells were gated on live cells and then on CD45^+^ (**Supplementary Figure 1**). The main cell subsets (CD3^+^, CD19^+^, CD56^+^, CD66b^+^, CD14^+^, and CD16^+^ cells) were analyzed within the CD45^+^ cells. Progressive gating on CD3^+^ T cell subsets was used to identify T cell subsets: CD8^+^, CD4^+^, and γδ T cells. T regulatory cells were identified as CD4^+^CD25^+^CD127^-^ cells within the gated CD4^+^ cells.

### 2.3 Statistical analysis

Statistical analyses were performed using GraphPad Prism 10 statistical software (GraphPad Software, San Diego, CA, http://www.graphpad.com). Demographic and clinical characteristics (e.g., BMI, estrogen duration, cycle day, endometrial thickness, infertility type) were compared between patients with a live birth and patients that failed implantation using Fisher’s exact test for categorical data and Wilcoxon two-sample tests for continuous variables. Immune cell differences between groups were assessed using appropriate two-group (unpaired) comparisons. Data normality was evaluated using the Shapiro–Wilk test. Normally distributed variables were analyzed using the unpaired Student’s t-test, while non-normally distributed variables were compared using the Mann–Whitney U test. P<0.05 was considered statistically significant. P values are indicated and the symbol ** indicates p values <0.01, while ns indicates not significant.

Receiver Operating Characteristic (ROC) curves are a statistical tool used to evaluate the accuracy of a diagnostic test by identifying the optimal cutoff value for distinguishing between two conditions—in this case, predicting live birth vs. implantation failure. We also calculated the area under the curve (AUC) to assess the overall accuracy of the test and cut off values; p<0.05 was considered statistically significant.

## 3 Results

### 3.1 The study cohort characteristics

The study cohort comprised 48 subjects, as detailed in **Table 1**. Out of 48 subjects, 30 (62.5%) had implantation after FET. Subsequently there were 24 live births (50%), 4 miscarriages (8.3%) and 3 biochemical pregnancies (6.3%). There were no ectopic pregnancies in our cohort.

**Table 1:** Patient demographics.

Characteristic	Live birth group n=24	Non-pregnant group n=17	P value
Age (years)	35.17 +/- 4.57	33.71 +/- 4.38	0.31
BMI (kg/m^2^)	27.91 +/- 6.85	27.24 +/- 7.45	0.77
Days on estrogen	15.00 +/- 3.53	15.00 +/- 4.75	0.97
Cycle day starting progesterone	13.63 +/- 2.68	12.71 +/- 1.53	0.21
Endometrial thickness at start of progesterone (mm)	10.63 +/- 1.69	10.24 +/- 1.44	0.44
PGT-A n (%)	15 (62.5%)	13 (76.5%)	0.50
Single embryo transfer n (%)	24 (100%)	17 (100%)	1.00
Etiology of infertility n (%)			0.08
Endometriosis	1 (4.2%)	1 (5.9%)	
Low ovarian reserve	3 (12.5%)	0	
Unexplained	6 (25.0%)	7 (41.2%)	
Male factor	5 (20.8%)	3 (17.6%)	
Polycystic ovarian syndrome	2 (8.3%)	2 (11.8%)	
Tubal	7 (29.2%)	3 (17.6%)	
Other	0	1 (5.9%)	

Data values are provided as n (%) or mean +/- standard deviation for continuous traits.

Patients with a live birth (LB) (n=24) were compared to those that did not achieve implantation and therefore did not achieve pregnancy (NP) after FET (n=17). There were no significant differences in age (35.17 ± 4.57 vs. 33.71 ± 4.38 years, p=0.31) or BMI (27.91 ± 6.85 vs. 27.24 ± 7.45 kg/m², p=0.77) between the two groups. The duration of estrogen treatment and cycle day at the start of progesterone were also similar between groups (15.00 ± 3.53 vs. 15.00 ± 4.75 days, p=0.97; 13.63 ± 2.68 vs. 12.71 ± 1.53, p=0.21, respectively). Endometrial thickness at the start of progesterone did not significantly differ (10.63 ± 1.69 mm vs. 10.24 ± 1.44 mm, p=0.44). PGT-A was performed in 62.5% of LB pregnancies compared to 76.5% in the NP group (p=0.50). Notably, all patients underwent single embryo transfer.

The etiology of infertility showed a trend toward significance (p=0.08). Among the LB cohort, the most common causes were tubal factor (29.2%), unexplained infertility (25.0%), and male factor (20.8%). In contrast, unexplained infertility was the most prevalent cause in the NP group (41.2%), followed by tubal factor (17.6%) and male factor (17.6%). Endometriosis and polycystic ovarian syndrome (PCOS) were comparable between groups (4.2% vs. 5.9% for endometriosis, and 8.3% vs. 11.8% for PCOS, respectively). One patient (5.9%) in the NP group had infertility classified as other due to a carrier of a monogenic disease undergoing PGT-M, while none were reported in the LB group.

### 3.2 Expression of immune cells in the uterine fluid

**Figure 1:**
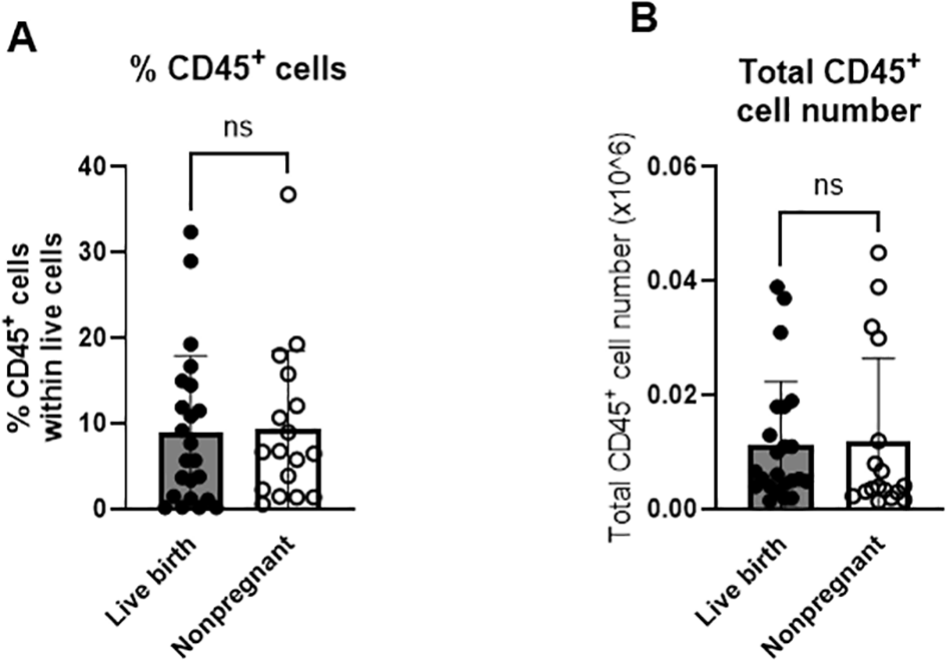
Immune cells in uterine fluid are equally distributed between the live birth group and non-pregnant group. Uterine fluid samples were collected on the day of embryo transfer, and pelleted cells stained with live/dead kit and anti-CD45, and analyzed on FlowJo software. (n=40 +/- SD unpaired t-test (two-tailed), *p<0.05). **(A)** percent (%) of CD45^+^ cells within all live cells **(B)** total number of CD45^+^ cells within all live cells.

Our previous study (26) was the first study to analyze composition of immune cells in the uterine fluid at the time of FET without pregnancy outcome (**Figure 1**). Here, we confirmed (in the bigger cohort including 48 subjects), that live, CD45^+^ cells are present in the uterine fluid at the time of FET and constitute around 9% of all viable uterine cells collected. We did not observe a statistically significant difference in the percentage and number of CD45^+^ cells between LB and NP groups (8.9% +/-9.0 SD vs. 9.3% +/- 9.2 SD p=0.621; 0.0113 x 106 +/- 0.0110 SD vs 0.0118 x 106 +/- 0.0146 SD p=0.359) (**Figure 1**).

### 3.3 Comparison of uterine fluid immune cell distribution between live birth and non-pregnant groups

**Figure 2:**
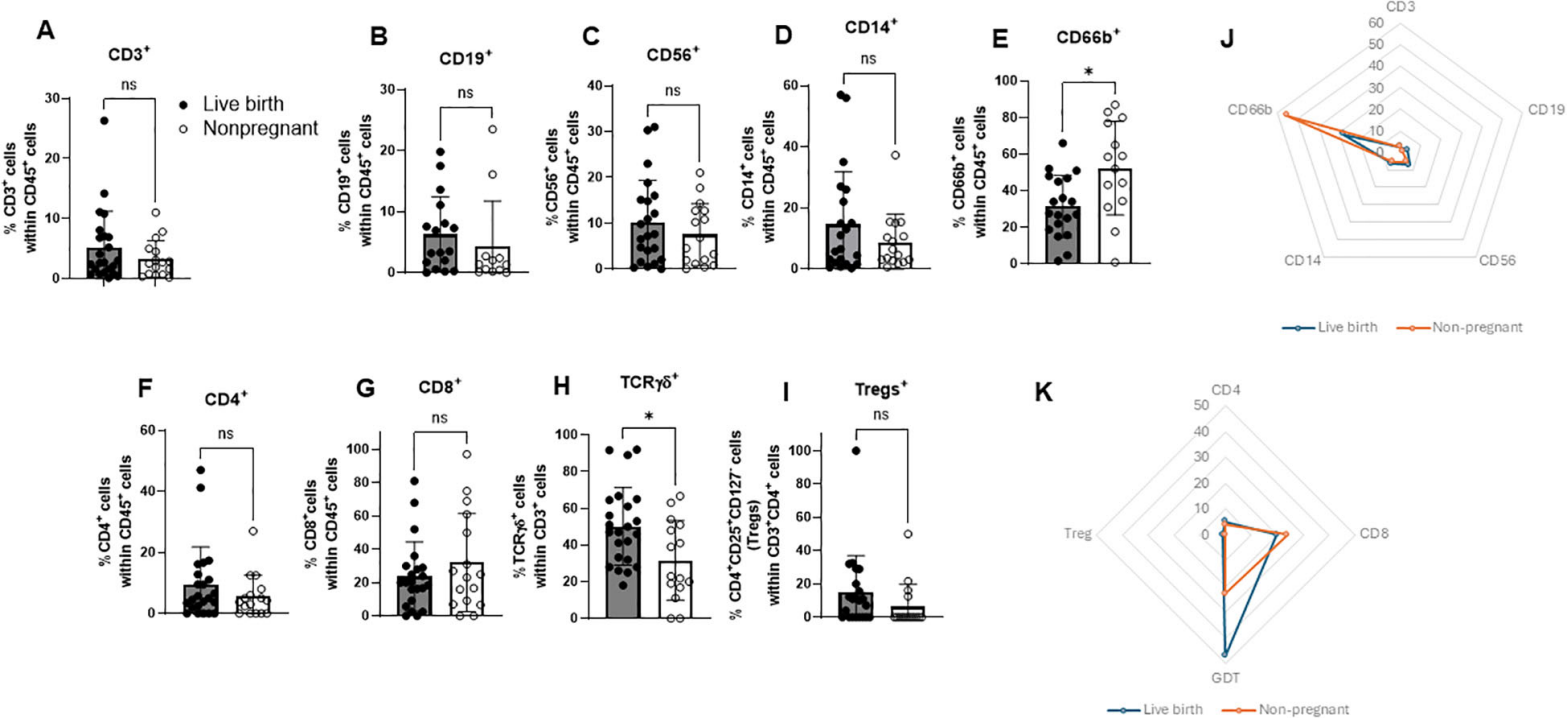
Comparison of surface immune markers between the live birth group and non-pregnant group. Uterine fluid samples were collected on the day of embryo transfer, and pelleted cells stained with live/dead kit and anti-CD45, anti-CD3, anti-CD4, anti-CD8, anti-TCR γδ, anti-CD25, andt-CD127, anti-CD19, anti-CD56, anti-CD14, anti-CD66b, and analyzed on FlowJo software. **(A)** Percent of CD3^+^**(B)** CD19^+^**(C)** CD56^+^**(D)** CD14^+^**(E)** CD66b^+^ CD45^+^ cells **(F)** Percent of CD4^+^**(G)** CD8^+^**(H)** TCR γδ^+^ within CD3^+^ cells and **(I)** percent of Tregs (CD25^+^CD27^-^) cells within CD3^+^CD4^+^ cells in live birth and non-pregnant group (n=40 +/- SD unpaired t-test (two-tailed), *p<0.05) **(J)** The radar chart summarizes distribution of immune cells in live birth group (blue line) and non-pregnant group (orange dotted line) (median values are plotted) within CD45+ cells **(K)**. The radar chart summarizes distribution of T cells in live birth group (blue line) and non-pregnant group (orange dotted line) (median values are plotted) within CD3+ cells.

To explore the relationship between various immune cells at the time of FET and reproductive outcomes, subjects entering the study were divided according to their pregnancy outcome (live birth and non-pregnant). There were no significant differences in baseline data between the two groups (**Table 1**). Analyzing the expressions of different immune-cell markers using flow cytometry, we observed a significant increase in the percentage of γδ TCR^+^ cells (γδ T cells) in the LB group compared to the NP group (50.15% +/-21.19 SD vs 31.64% +/-21.72 SD; p=0.0197) (**Figure 2H**). In contrast, the percentage of CD66b^+^ cells (neutrophils) was increased in NP group compared to LB group (52.36% +/- 25.62 SD vs 31.44% +/-16.97SD; p=0.0143) (**Figure 2E**). The radar chart shows a summary of the distribution of different immune cells in LB and NP groups, where the dotted orange line represents distribution of the NP group and blue line represents the LB group, **Figures 2J, K**.

There was no statistically significant difference in percentage of CD3 (5.23% +/- 6.02 vs 3.27% +/-3.06; p=0.136), CD19 (6.27% +/-6.13 SD vs 4.22% +/- 7.49 SD; p=0.136), CD56 (10.08% +/-9.28 SD vs 7.50% +/-6.77 SD; p=0.468), CD14 (14.60% +/- 17.15 SD vs 8.79% +/- 9.16 SD; p=0.688), CD4 (9.51% +/- 12.27 SD vs 5.59% +/- 7.01 SD; p=0.327), CD8 (23.68% +/-20.78 SD vs 32.10% +/- 29.54; p=0.563), and CD4^+^CD25^+^CD127^-^ (14.76% +/- 22.16 SD vs 6.27% +/-13.56; p=0.059) between LB and NP groups (**Figures 2A–D, F–I**).

### 3.4 Uterine γδ TCR^+^ and CD66b^+^ cells as potential biomarkers for predicting pregnancy outcomes after FET through the analysis of Receiver Operating Characteristic curves

**Figure 3:**
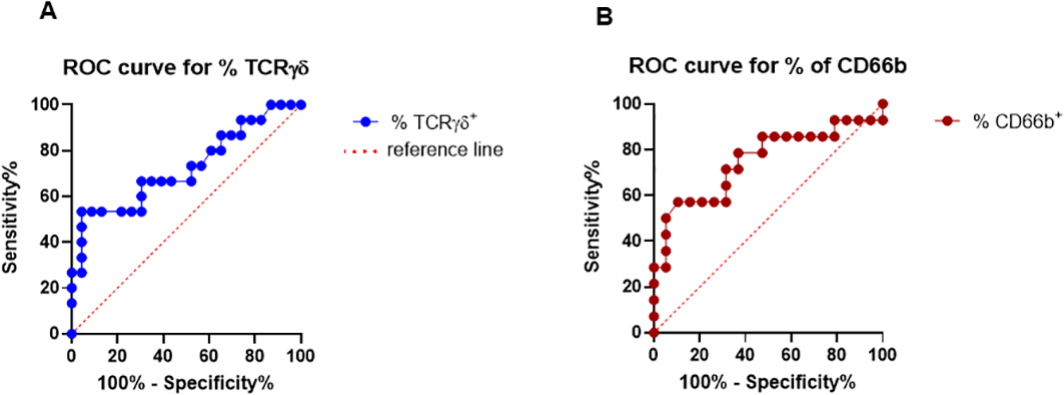
Evaluation of percentage of uterine TCR γδ^+^ and CD66b^+^ cells by ROC curve analysis as a novel diagnostic pregnancy-predictor test for FET. ROC (receiver operating characteristics) curve presents average values of the sensitivity for a % of TCR γδ^+^ and CD66b^+^cells over all possible values of specificity or vice versa.

We analyzed percentages of γδ TCR^+^ and CD66b^+^ cells in the uterine lining of patients undergoing FET. ROC analysis showed that the optimal cutoff value for γδ TCR^+^ cells is >24% and classify subjects below this value as a higher risk for not getting pregnant using the derivation set. Specificity for this value is very high, 95%, meaning that only 5% of pregnant patients will have less than 24% of uterine fluid γδ TCR^+^ cells. To quantify the overall ability of the uterine fluid cell-frequency test to discriminate between those individuals that will achieve a LB and those without pregnancy, we used area under a ROC curve (AUC) statistic. We found an AUC of 0.72 with 95% confidence interval (CI) 0.5504 to 0.8989 for γδ TCR^+^ cells, which represents the probability of 72% that a randomly selected non-pregnant patient undergoing FET will have a lower test result than a randomly selected patient with a LB. Importantly, higher AUC indicates better predictive ability with p=0.0206 which overall confirms that our test discriminates between LB and NP, **Figure 3**.

We have also found that optimal cutoff value for CD66b^+^ cells is >39.60% and classify patients above this value as a higher risk for implantation failure with specificity of 68.42% (95% CI 46.01% to 84.64%). In addition, we found that AUC is 0.75 (95% CI 0.5681 to 0.9319) with p= 0.0154, confirming overall ability of CD66b test to discriminate between NP and LB, with a probability of 75% that a randomly selected NP patient undergoing FET will have a higher CD66b test result than a randomly selected patient with a LB at the time of the FET, **Figure 3**.

Overall, our study is the first study to present the potential of immune cell profiles at the time of FET using the index cycle as a predictive tool for IVF outcomes, highlighting the importance of uterine immune responses in pregnancy success.

## 4 Discussion

Embryo implantation is a highly regulated process that occurs during a limited window of uterine receptivity (27). Many studies have focused on analyzing endometrial tissue to uncover the molecular mechanisms essential for successful implantation. However, obtaining tissue samples through biopsy can be invasive and may disrupt the implantation process. Additionally, collecting endometrial tissue at the incorrect time can lead to misinterpretation of the molecular characteristics specific to the implantation site. Overall, the current endometrial receptivity diagnostic tests are overly simplistic and have limited clinical utility (28). Our study demonstrates that assessment of endometrial immune composition during the embryo transfer cycle is feasible without measurably altering the endometrial environment and identifies γδ T cells (TCR γδ^+^ T cells) and neutrophils (CD66b^+^ cells) as immune cell subsets associated with pregnancy outcome. While these findings suggest that endometrial immune profiles may contribute to implantation success and live birth, they should be interpreted as hypothesis-generating, and are not intended to inform clinical decision-making regarding embryo transfer timing or immunomodulatory interventions at this stage.

Previous studies have highlighted the importance of interplay between endometrial receptivity/selectivity index and embryo quality in successful implantation (29, 30). Our exploratory study is aiming at finding this window of optimal endometrial milieu that will foster implantation and improving live birth following FET. Several factors influence successful implantation in FET cycles, including immune cell profiles in the uterus. All immune features described in this study are reported as associated parameters and should not be interpreted as validated biomarkers or indicators of clinical readiness. Our study found statistically significant differences in the percentage of γδ T cells and CD66b^+^cells in the uterine fluid between LB and NP subjects with high specificity of 95% and 68.4%, respectively (**Figure 2**). We also found we could increase the specificity of our CD66b^+^ to 95% by lowering the cut off to 55.25%. These findings indicate that variation in these two cell populations at the time of FET is associated with subsequent pregnancy outcomes, warranting further investigation.

Our study found that endometrial γδ T cells at the time of embryo transfer are significantly more abundant in patients with a LB. We identified a threshold of 24% γδ T cell expression in the endometrium, above which patients are more likely to have a successful pregnancy. γδ T cells are unique in that they bridge both the innate and adaptive immune systems, producing cytokines that play a key role in immune regulation and protecting the placenta. They are known to recognize alloantigens without MHC activation, which is critical for maintaining immune tolerance during pregnancy, as the syncytiotrophoblast (the layer between maternal and fetal tissues) lacks HLA expression (31, 32). γδ T cells also secrete anti-inflammatory cytokines, such as IL-10 and TGF-β, which support placental function and embryo implantation by promoting the invasion of extravillous trophoblasts (33–37). Additionally, γδ T cells contribute to tissue repair and homeostasis, aiding in the protection of the fetus and the placenta (38, 39). These findings suggest that a higher percentage of γδ T cells could be a positive predictor of pregnancy success, while lower levels may be linked to pregnancy failure. The increased percentage of γδ T cells, contributing to more anti-inflammatory cytokine milieu, is consistent with the results of one of the current authors’ studies evaluating serial IL-10 and TNF-α concentrations in first trimester of pregnancy. IL‐10 concentrations in normal pregnancies were significantly higher than in pregnancies ending in a loss starting at 6‐8 weeks of gestation. Conversely, TNF-α concentrations were significantly lower in normal pregnancies than in pregnancies that ultimately ended in a pregnancy loss starting at 3‐5 weeks of gestation. The IL‐10 to TNF-α ratio in normal pregnancies was significantly higher from 4 to 9 weeks compared to ultimately nonviable pregnancies (P <.05) (40). It is intriguing that one of the predominant cell subsets in the decidua, dNK cells, are not the predominant cell subset in the uterine fluid (**Figure 2C**). Our explanation is that dNK cells might have highly regulated expression of the adhesion molecules that are not allowing “exit” of dNK cells from the tissue (endometrium) into intraepithelial compartment (lumen of the uterus). Further research is needed to clarify the full role of dNK cells and γδ T cells in successful pregnancy.

Our study reveals that neutrophils (CD66b^+^) are significantly more abundant at the time of FET in patients who did not achieve implantation. We identified a cutoff value of >55.25% neutrophils, above which patients are at higher risk of not becoming pregnant. Neutrophils, which typically make up 50-70% of leukocytes in peripheral circulation (41), constitute about 42-48% of uterine fluid leukocytes (26). These cells are key players in the immune response to infections and inflammation. While their role in implantation and pregnancy is not fully understood, studies suggest they are involved in tissue remodeling and decidualization in early pregnancy (42). Increased neutrophil presence has been linked to normal pregnancies (42), but also to pathologies like pregnancy loss, preterm labor, and preeclampsia (43–51). Our findings suggest that an elevated neutrophil count could be associated with complications of implantation and pregnancy success. Further research is needed to understand the exact mechanism behind this relationship.

Mechanistically, our findings fit in the established immunological framework of endometrial receptivity where the opposing association of neutrophils and γδ T cells with reproductive outcomes suggests that the balance between inflammatory and regulatory innate immune populations at embryo transfer is critical. Excess neutrophil-driven inflammation may overwhelm tolerogenic pathways, whereas γδ T cell enrichment may actively restrain inflammation and facilitate maternal–fetal immune adaptation. This concept aligns with emerging evidence that implantation failure reflects not immune quiescence but rather immune dysregulation, characterized by exaggerated innate activation and insufficient regulatory control (2). Future studies should focus on functional profiling of γδ T cell subsets and neutrophil activation states, as well as longitudinal analyses to determine whether modulating these pathways can restore endometrial receptivity.

A balanced discussion of the strengths and limitations of this study is essential to contextualize its findings and guide future research in the field. Our work’s most distinguishing feature is the novel noninvasive technique of sample acquisition using the same catheter that was used for the embryo transfer. This technique allows sampling from the exact window of implantation of intended cycle for transfer and ultimately can give way to the development of a POCT which will allow the physician to decide whether to perform the transfer based on the current immune environment. The goal is to produce a clinically interpretable immune profile within a time window that still allows modification of the treatment strategy before embryo transfer. The resulting immune profile would directly inform individualized clinical decisions, including: immunomodulatory treatment selection (corticosteroids, low-dose tacrolimus, or other immunoregulatory agents), supportive therapies that enhance endometrial receptivity (e.g., G-CSF, PBMC-derived treatments, or experimental ILC-targeted approaches) (12, 13, 15, 52–56). Clinicians could opt to defer embryo transfer, allowing time to correct the immunological imbalance before attempting implantation or tailoring luteal-phase support or peri-transfer interventions, such as modifications in progesterone support, the addition of anti-inflammatory agents, or protocols designed to promote regulatory immune pathways at the maternal–fetal interface.

Furthermore, our study was able to analyze the immune environment of the uterus without obtaining an endometrial biopsy, as is typically done in investigations of the endometrium. Endometrial biopsies are invasive and painful for patients, and the results may not be applicable to future cycles, therefore assessment of the endometrial milieu using the transfer catheter is a preferred method. Finally, our work established thresholds of γδ T cells and neutrophils necessary to predict LB and no implantation, respectively, which has never been established. Accordingly, the present work is intended to be exploratory and hypothesis-generating, with observed immune cell associations interpreted descriptively and without implication of causality, biological validation, or clinical utility. The limitations include our small sample size which may limit the power of our statistical analysis. We believe by expanding our study population we will improve the specificity and sensitivity of our cut off values for both the TCR γδ^+^ and CD66b^+^ cell subtypes. In addition, our sampling technique uses an embryo transfer catheter with an outer sheath and an inner catheter. Although only the inner catheter is used for the study, it cannot be excluded that cells originating from the endocervix may be present in the sample. Furthermore, while subjects denied any prior diagnoses of inflammatory conditions upon initial evaluation and were not on corticosteroids or anti-inflammatory medications, the presence of subclinical inflammatory alterations in the endometrium cannot be excluded.

Future work will focus on expanding cohort size and incorporating higher-dimensional immune profiling approaches, such as multicolor flow cytometry, to further characterize endometrial immune cell composition. These efforts aim to contribute to a more detailed understanding of the endometrial immune milieu across embryo transfer cycles. While such studies may inform the development of analytical or methodological tools for immune profiling, any translation toward point-of-care applications or clinical use will require substantial technical development and prospective validation and is beyond the scope of the present work.

## Data Availability

The raw data supporting the conclusions of this article will be made available by the authors, without undue reservation.
