# Supplementary material for: Endometrial immune cell profile at the time of frozen embryo transfer as prognostic indicator of live birth
Source: Front Immunol. 2026 Mar 6;17:1719211. doi: 10.3389/fimmu.2026.1719211 (PMC13002432; doi:10.3389/fimmu.2026.1719211)
Supplement: Supplementary file 1 [file DataSheet1.pdf]

**Supplementary Figure 1.**

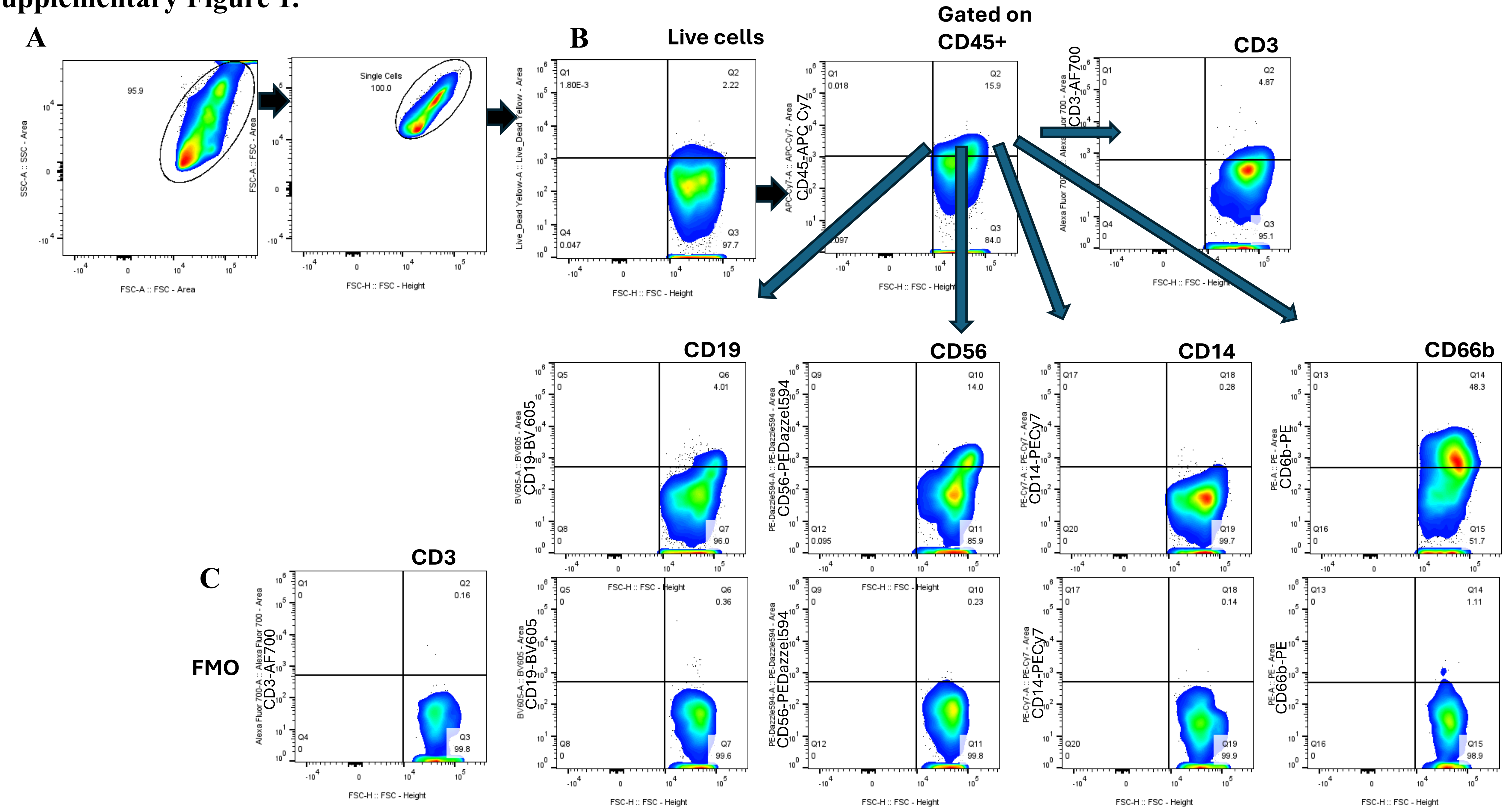

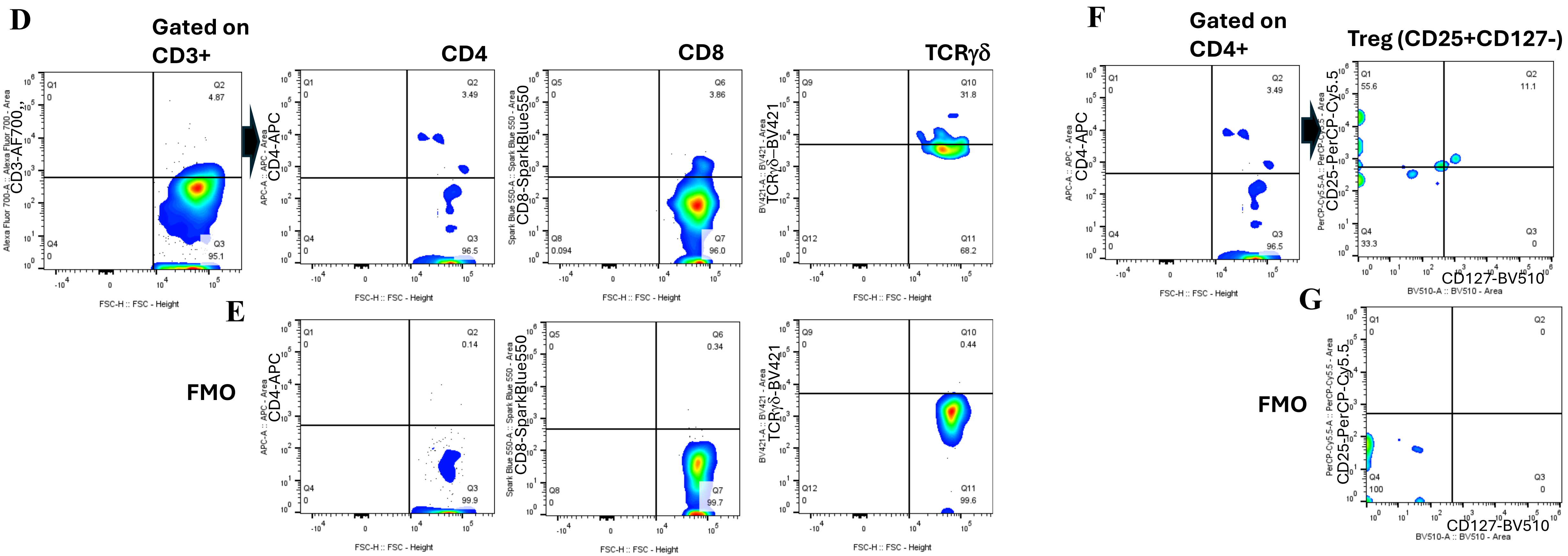


**Supplementary Figure 1. Flow cytometry gating strategy for immune cells present in the uterine fluid on the day of the embryo transfer.** Representative flow cytometry plots and immune subsets gating strategy for uterine cells collected from females who underwent FET. Uterine cells were stained with live/dead kit first and then with the following surface markers: CD45, CD3, CD19, CD56, CD14, CD66b, CD4, CD8, TCRγδ, CD25, CD127, acquired on Sony SP6800 Spectral Analyzer, and analyzed on FlowJo software. A) All acquired cells were first analyzed for size (FSC) and granularity (SSC) and single cells (FSC-area vs FSC-height) were included in further analysis B) Live single cells were gated and analyzed for expression of CD45 and subsequently live/CD45+ cells were analyzed for expression of CD3, CD19, CD56, CD14 and CD66b. D) In addition, gated CD3+ cells were analyzed for expression of CD4, CD8 and TCRγδ. F) Gated CD4+ T cells were analyzed for the expression of CD25 and CD127. Fluorescence minus one (FMO) included samples that were stained for C) live/dead and CD45; E) live/dead, CD45 and CD3 and G) live/dead, CD45, CD3, and CD4
